# A comparison of pre- and post-operative outcomes in living donors undergoing transperitoneal laparoscopic nephrectomy and open nephrectomy: a retrospective single-center study

**DOI:** 10.1590/1516-3180.2022.0488.R1.070723

**Published:** 2023-12-08

**Authors:** Ahmet Keleş, Cevdet Kaya

**Affiliations:** I MD. Urologist, Department of Urology, School of Medicine, Istanbul Medeniyet University, Uskudar, Turkey.; II MD. Professor of Urology, Department of Urology, School of Medicine, Marmara University, Istanbul, Turkey.

**Keywords:** Kidney, Transplantation, Laparoscopy, Donor nephrectomy, Laparoscopic renal surgeries, Live donor

## Abstract

**BACKGROUND:**

Kidney transplantation is often regarded as the preferred therapy for end-stage renal disease. Several surgical procedures have been developed to reduce postoperative donor complications, while maintaining kidney quality.

**OBJECTIVE:**

This study aimed to compare the preoperative and postoperative outcomes of living kidney donors who underwent either transperitoneal laparoscopic nephrectomy or open nephrectomy.

**DESIGN AND SETTING:**

Retrospective study conducted in Istanbul, Turkey.

**METHODS:**

Fifty-five living-related kidney donors underwent nephrectomy and were retrospectively divided into two groups: 21 donors who underwent open nephrectomy (Group 1) and 34 donors who underwent transperitoneal laparoscopic nephrectomy (Group 2).

**RESULTS:**

In comparison to the donors who underwent open nephrectomy, those who underwent transperitoneal laparoscopic nephrectomy had significantly shorter postoperative hospital stays (2.3 ± 0.2 versus 3.8 ± 0.8 days, P = 0.003), duration of urinary catheterization (1.2 ± 0.8 days versus 2.0 ± 0.7 days, P = 0.0001), operating times (210 ± 27 minutes versus 185 ± 24 minutes, P = 0.02), and less blood loss (86 ml versus 142 ml, P = 0.048). There was no statistically significant difference between the two groups with regard to the estimated blood transfusion and warm ischemia time. The preoperative week, first postoperative week, and 1-month postoperative serum creatinine levels were comparable between the groups.

**CONCLUSIONS:**

Laparoscopic donor nephrectomy can be safely performed at centers with expertise in laparoscopic surgery. Laparoscopic donor nephrectomy has better outcomes than open donor nephrectomy in terms of length of hospital stay, duration of urinary catheterization, operating time, and blood loss.

## INTRODUCTION

Improvements in technique have resulted in better outcomes for laparoscopic donor nephrectomy.^1,2^ Ratner introduced this technique in 1995; it is now performed more frequently, with higher success rates.^3^ Donor nephrectomy is distinguished from other surgical procedures in that the surgery is performed on a healthy individual to improve the health of another. This places a strong emphasis on reducing donor morbidity and implementing minimally invasive approaches. There is an increasing gap between organ supply and demand, which has also played a role in the recent trend toward living-donor kidney transplantation.

Transplant recipients receive numerous benefits from living donations, and the operation can be planned. However, donors do not receive the same benefits.^4^ Some of the advantages of transperitoneal laparoscopic nephrectomy over open methods are reduced intraoperative blood loss, improved aesthetics, shorter hospital stay, and faster overall postoperative recovery, which allows the recipient to return to normal activity in a shorter period. As a result of these advantages, the number of living-donor kidney transplants has increased.^5,6^ Currently, most transplantation centers harvest living-donor kidneys using a conventional laparoscopic surgical approach.^6^ Transplantation teams accept living kidney donations under conditions that suggest a safe long-term outcome for the donor.^7^

## OBJECTIVE

We aimed to evaluate and compare early complications and renal function following donor nephrectomy performed by an experienced surgeon using either an open or laparoscopic approach.

## METHODS

### Patients

This study included 55 living-related kidney donors who underwent nephrectomy between March 2010 and March 2014. Twenty-one of these patients underwent open nephrectomy (Group 1), and 34 underwent laparoscopic nephrectomy (Group 2). Donors were interviewed regarding their surgical preferences, which included both open and laparoscopic donor nephrectomies. Patients aged between 18 and 75 years with end-stage renal disease, (defined as an estimated glomerular filtration rate of < 20 mL/min, symptomatic uremia, or dialysis necessity), who received an organ from a live donor from their family were included in the study. The exclusion criteria were nephrectomy of cadaver origin, a follow-up duration less than 1 month, and pre- or post-operative contrast-enhanced imaging. Patient data were collected from the hospital's medical records database and through patient interviews.

The donations were voluntary and in accordance with the Human Organ Transplant policies and regulations in Turkey. This study was approved by the Ethics Committee of the Marmara University School of Medicine (ID: 11.09.2014/15/14, date: 07.11.2014).

### Evaluation of donors

A detailed assessment of the donors is routinely performed to ensure long-term safety. According to the United Network for Organ Sharing (UNOS), follow-up and monitoring of serum creatinine levels are required after a post-donation duration of at least 2 years. The functional performance of the kidney is mainly evaluated using the best overall measure: the glomerular filtration rate (GFR). The Modification of Diet in Renal Disease (MDRD) formula was used to calculate and perform a detailed evaluation of pre- and post-operative kidney function using estimated glomerular filtration rates (eGFRs). This formula was developed by the MDRD study group.^8^ The ability of the MDRD formula to predict GFRs was analyzed by comparing the results obtained from other prediction equations of healthy participants without any known kidney disease.^9^ Age, sex, and serum creatinine levels were recorded for the study participants to estimate GFR using the abbreviated version of the MDRD formula:^10^


$$\text{eGFR}\left(\text{ml/}\min/1.73{\text{m}}^{2}\right)=175\times {\left({\text{S}}_{\text{cr}}\right)}^{-1.154}\times {\left(\text{age}\right)}^{-0.203}\times \left(0.742\text{if female}\right).$$

### Operative procedures

Before the procedures, the donors and recipients underwent a comprehensive medical assessment, and light bowel preparation was performed before surgery. The renal vessel anatomy of all donors was evaluated using abdominal computed tomography (CT) imaging.

The best use of renal vein length was achieved by left donor nephrectomy, which was routinely performed. A retroperitoneal flank incision was used for classic open nephrectomy. Transperitoneal laparoscopic nephrectomy was performed on the left side with the patient in the decubitus position. The procedure was performed using a video laparoscope and dissecting instruments. The procedure started with the inflation of the abdomen using a Verres needle. The abdominal cavity was inspected to ensure that there was no damage after inserting a 12-mm trocar. Two additional trocars were then inserted, the first superolateral to the umbilicus and the second at the midline of the rib cage. We often preferred using 10-mm trocars because they were easy to interchange with laparoscopic instruments. Dissection started with a Toldt line incision and reflection of the descending colon and continued until Gerota's fascia was seen. The medial gonadal vein was observed, and the dissection was traced up to the renal hilum. The renal artery and gonadal, renal, and adrenal veins were then carefully dissected and transected. The progression of the level of the iliac vessels was made by ureteral dissection, and the ureter was transected distally. Each of the renal arteries, veins, and ureters were stapled across before the kidney could be removed from the bag. In the final step, the completely freed kidney was removed from the Gibson incision.

### Statistical analysis

The data were analyzed for frequencies, and the chi-square test was used to compare categorical variables. The mean values of the numerical variables between the groups were compared using the Mann–Whitney U test. SPSS for Windows (version 20.0; SPSS Inc., Chicago, Illinois, United States) was used for the statistical analysis of all data. Statistical significance was set at P < 0.05.

## RESULTS

Donor demographics, estimated blood loss, operative characteristics, mean hospital stay, mean operative time, warm ischemia time of the graft, number of vessels, reduction rate of donor serum creatinine levels in the first seven days and one month after renal transplantation, and donor complications were compared between the two surgical approaches.

**Figure 1:**
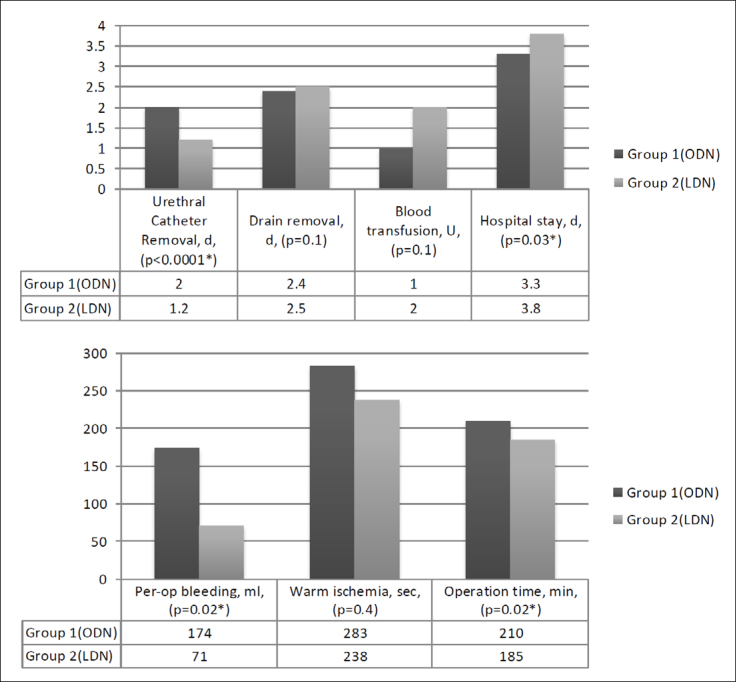
Intraoperative and postoperative parameters.

Living-donor nephrectomies were performed on all 55 donors (34 transperitoneal laparoscopic nephrectomies; 21 open nephrectomies). The donor demographics and indications for surgery were similar in both groups. A comparison of donor characteristics is shown in Table 1. Abdominal CT angiography revealed the presence of double renal arteries in two of the 21 donors undergoing open nephrectomy (Group 1) and three of the 34 donors undergoing transperitoneal laparoscopic nephrectomy (Group 2). The mean warm ischemia time was 283 ± 152 s for open nephrectomy and 238 ± 73 s for transperitoneal laparoscopic nephrectomy (P = 0.4). In comparison to the donors who underwent open nephrectomy, the donors who underwent transperitoneal laparoscopic nephrectomy had a significantly shorter postoperative hospital stay (2.3 ± 0.2 versus 3.8 ± 0.8 days, P = 0.003), duration of urinary catheterization (1.2 ± 0.8 versus 2.0 ± 0.7 days, P = 0.0001), operating time (210 ± 27 versus 185 ± 24 minutes, P = 0.02), and significantly less blood loss (86 ml versus 142 ml, P = 0.048) (Table 2). There was no statistically significant difference in the estimated blood transfusion and warm ischemia time between the two groups (Figure 1). There were no cases of graft loss or conversion from laparoscopic to open surgery. Two patients in Group 1 had fevers > 101.5°F due to atelectasis, which was treated with intravenous antibiotics. In the laparoscopic group, one donor had a pneumothorax that required thoracic drain tube placement, and a small umbilical hernia developed at the hand port site.

**Table 1:** Patient characteristics

Parameter		Group 1 (ODN)	Group 2 (LDN)	P value
Patient, (n)		21	34	
Age (mean), SD, (years)		45 ± 9.6	45 ± 8.9	0.7
Gender, (n)				
	Male	8	12	0.7
	Female	13	22	
Laterality, (n)				
	Right	2	0	0.1
	Left	19	34	
Renal artery, (n)				
	Single	19	31	0.9
	Double	2	3	
BMI (mean), SD, (kg/m^2^)		29.7 ± 4.5	27.1 ± 4.2	0.5

Mann-Whitney U and Chi-Square tests used.

SD = standard deviation; BMI = body mass index; ODN = open donor nephrectomy; LDN = laparoscopic donor nephrectomy.

**Table 2:** Clinical and laboratory data of the groups

Parameter	Group 1 (SD)	Group 2 (SD)	P value
Preoperative Cre (mg/dl)	0.72 (0.14)	0.75 (0.15)	0.6
Postoperative 1st week Cre (mg/dl)	1.02 (0.23)	1 (0.23)	0.9
Postoperative 1st month Cre (mg/dl)	1.02 (0.24)	1.06 (0.20)	0.5
Preoperative microalbumin (mg/dl)	4.9 (2.93)	2.1 (1.7)	0.19
Postoperative microalbumin (mg/dl)	11.2 (6.69)	2.1 (1.9)	**0.03**
Urethral catheter removal (day)	2 (0.7)	1.2 (0.81)	**< 0.0001**
Drain removal (day)	2.4 (0.75)	2.5 (2.08)	0.1
Blood transfusion (U)	1	2	0.1
Perioperative bleeding (ml)	174 (142)	71 (61)	**0.02**
Warm ischemia time (sec)	283 (152)	238 (73)	0.4
Operation time (min)	210 (27)	185 (39)	**0.02**
**Hospital stay (day)**	**3.8 (0.85)**	**2.3 (0.2)**	**0.003**

Continuous data presented as mean + standard deviation (SD).

Cre = creatinine; SD = standard deviation; U = unit; Min = minutes; Sec = second.

The creatinine levels in the preoperative week and first postoperative week and month were comparable between the two groups (Table 2). The mean eGFRs preoperatively and at postoperative week 1 and month 1 were comparable between the two groups. A statistically significant reduction in eGFR was noted at postoperative week 1.

**Figure 2:**
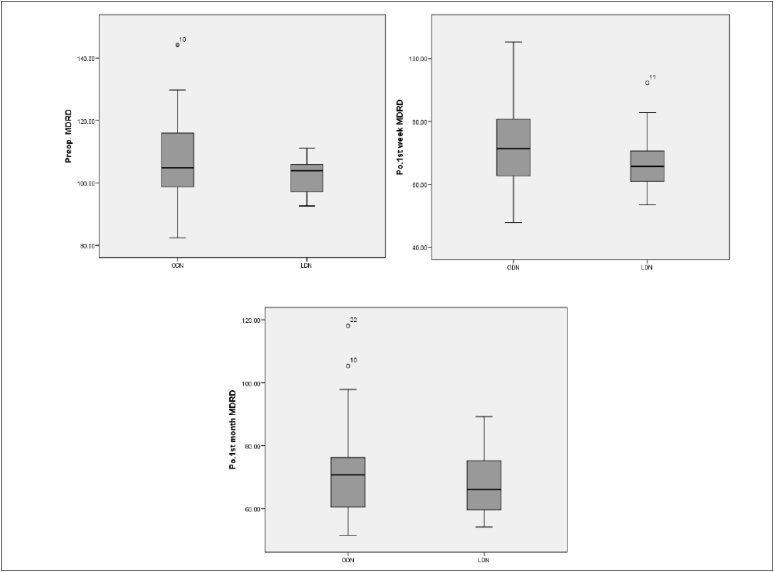
Comparison of the mean Modification of Diet in Renal Disease (MDRD) preoperatively and at postoperative week 1 and month 1.

**Figure 3:**
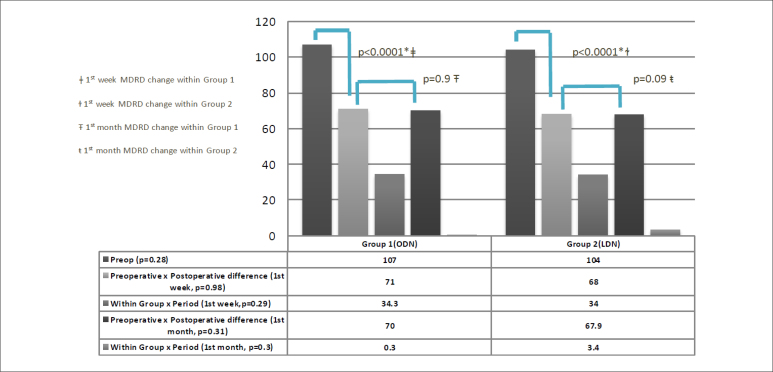
Comparison of the groups in terms of estimated glomerular filtration rates (eGFR) using the Modification of Diet in Renal Disease (MDRD) formula.

The mean MDRD values in Group 1 and Group 2 were 107 ± 16.1 and 104.2 ± 14.2 ml/min/m^2^, respectively (P = 0.28). After postoperative week 1, the MDRD values decreased to 34 and 34.3 ml/min/m^2^ in Group 1 and Group 2, respectively (between-group comparison P = 0.98, within-group comparison P < 0.0001 for both groups) (Figures 2 and 3). At postoperative month 1, the MDRD values stabilized for donors in both groups.

A within-group assessment of the surgical learning curve was also performed for Group 2 (laparoscopic nephrectomy), which showed a significant reduction in surgery time following the first 10 cases of living donors (P = 0.0001). From an average of more than 200 min for the first 10 cases, surgery time was reduced to less than 200 min after the initial 10 procedures (249 ± 19 versus 197 ± 13 minutes). No statistically significant differences were found based on the learning curve between the two groups (i.e., the first 10 subjects and the remaining subjects) in terms of laboratory parameters, perioperative blood loss, analgesic requirement, and postoperative clinical parameters. No deaths occurred among the 55 donors included in this study.

## DISCUSSION

Limitations in the organ donor supply continue to pose a significant challenge to improving the outcomes of patients with end-stage renal failure. It has become imperative to expand the potential living-donor pool; this has been successfully achieved with the advent of laparoscopic donation because of the rapid recovery and return to normal activities.^11^

Living-donor nephrectomy is considered the most stressful intervention in urology because, by definition, it involves an altruistic organ donation by healthy individuals.^12^ Donor nephrectomy is a unique operation in that it exposes a person in complete health to the potential complications of major surgery for the benefit of the recipient. Therefore, donor safety should be the priority in kidney transplantation.^13^

The rate of live kidney donation in the United States, Europe, and Turkey has increased directly with the increased use of living transperitoneal laparoscopic nephrectomy.^14,15^ There is an association between the introduction of transperitoneal laparoscopic nephrectomy and the expansion of the living kidney donor pool in particular, and renal transplantation in general; evidence of this has been previously reported.^15^

However, morbidity and complication rates have been found to increase among surgeons just beginning to learn the technique for laparoscopic donor nephrectomy. To minimize the warm ischemia time, careful handling of the vessels and kidney, rapid specimen extraction, and extensive vascular dissection are required, and advanced laparoscopic skills are necessary. The operative time and complication rates were used to measure the surgical learning curve. After significant gains in experience, the incidence of delayed graft function and operative time decreased considerably. Leventhal et al. reported that the majority of complications occurred during the first 30 cases.^2^ Additionally, four of the five conversions occurred in the first 40 cases.^2^ The learning curve for laparoscopic nephrectomy flattened after 10 cases, even in the hands of an experienced laparoscopic surgeon. Based on the transperitoneal laparoscopic nephrectomy experience gained during this study, we adopted a point of view that seemed promising in terms of minimizing the morbidity associated with the learning curve. Moreover, from the recipients’ standpoint, the transperitoneal laparoscopic nephrectomy results were comparable to those obtained using the well-established open approach. From the donors’ standpoint, the transperitoneal laparoscopic nephrectomy results were superior to those of the open approach.

The increasing number of living donors has resulted in the need for more information about the potential risks of living with one kidney. Our findings indicate that most kidney donors have a favorable renal course. However, additional donors should be evaluated to confirm these findings. After donation, numerous donors developed increased serum creatinine levels, which may be associated with increased cardiovascular mortality.^16^ In a study by Berber et al., the postoperative serum creatinine levels were within normal limits.^17^ Therefore, the development of kidney dysfunction or failure in a donor is highly unlikely. Despite the limited follow-up and number of patients, several studies have examined changes in serum creatinine levels.^18^ Hartmann et al. reported 1,800 cases of living donors, of which only seven developed end-stage renal disease.^19^ Another study reported 402 cases of living donors in Sweden, and only one required hemodialysis due to postoperative renal failure.^20^ In our study, the donors did not develop end-stage renal disease in the long-term follow-up, a result consistent with the findings of previous studies.

During transperitoneal laparoscopic nephrectomy, minimization of the warm ischemia time is crucial to avoid renal injury. In one study, the reported warm ischemia time ranged between 2.6 and 6 min.^21^ In a study of 500 cases of laparoscopic donor nephrectomy reported by Leventhal et al., the average warm ischemia time was 2.6 min.^22^ Previous studies have reported shorter warm ischemia times for transperitoneal laparoscopic nephrectomy in comparison to open donor nephrectomy. In our study, the mean duration of warm ischemia was 2.7 min. Ideally, the warm ischemia time should not exceed 3 min in transplant surgery.^23^ In general, the warm ischemia time is expected to be shorter in minimally invasive donor nephrectomy than in open donor nephrectomy. However, in our study, we observed a longer warm ischemia time in the open donor nephrectomy group than that reported in the literature. Specific factors may have contributed to the longer warm ischemia time such as the complexity of the procedure, surgical team experience, or variations in the technique used. It is also possible that there are issues related to the preservation and handling of the kidney after removal that may affect the warm ischemia time. Another reason could be that open donor nephrectomy involves a larger surgical incision and more extensive dissection of the kidney and its blood vessels, which increases the risk of bleeding and prolongs the warm ischemia time. The longer operative time for open donor nephrectomy differed from that reported in the literature. This discrepancy may be because the duration of open donor nephrectomy varies depending on the surgeon's experience, the patient's anatomy, and the type of surgical technique used.

We found that the results from the donors’ standpoint corresponded with those reported in other studies that compared transperitoneal laparoscopic and open nephrectomy. These include shorter hospital stay, less blood loss, and similar rates of complications.^1,11,18,22^

Our study had some limitations. First, although the collection of laparoscopic data was prospective, this study was retrospective, and the majority of open nephrectomy data were historical. Consequently, a significantly longer follow-up period was observed in the open nephrectomy group (Group 1). Second, the higher American Society of Anesthesiologists status of the open nephrectomy group constituted a discrepancy between the two study groups that was unlikely to account for the longer hospital stay. Another limitation was the relatively small sample size of each group. An additional limitation was the inadequacy of our findings, which indicated a difference in the length of the donors’ hospital stay between the two groups. Additional outcomes, such as functional status and patients’ quality of life, could have been more detailed and informative.

## CONCLUSIONS

Transperitoneal laparoscopic living-donor nephrectomy is a less invasive approach than open nephrectomy. This has a significant influence on kidney donor operations. Consequently, donor morbidity decreased while a higher-quality allograft for the recipient was maintained. Transperitoneal laparoscopic donor nephrectomy can be safely performed in centers with expertise in laparoscopic surgery. From the donor's perspective, transperitoneal laparoscopic donor nephrectomy has better outcomes than open donor nephrectomy in terms of the length of hospital stay, duration of urinary catheterization, operating time, and blood loss.
